# The clinical effective of Baduanjin rehabilitation training on limb motor function, daily life, and quality of life in elderly patients with hemiplegia after stroke

**DOI:** 10.1097/MD.0000000000043495

**Published:** 2025-07-25

**Authors:** Hongyan Zhang, Rong Wan, Lihui Zhou, Kangping Zhu, Jing Zhang

**Affiliations:** a The Rehabilitation Neurology Department II of the 904th Hospital of the Joint Logistics Support Force, Wu xi, Jiangsu, China; b Quality Management Office, 904 Hospital, Joint Logistics Support Force, Wuxi, Jiangsu, China; c Department of Neurology, Affiliated Wuxi Fifth Hospital of Jiangnan University, Wuxi, Jiangsu, China; d The Rehabilitation Neurology Department II of the 904th Hospital of the Joint Logistics Support Force, Wu xi, Jiangsu, China; e Department of Nursing, 904 Hospital, Joint Logistics Support Force, Wuxi, Jiangsu, China.

**Keywords:** Baduanjin rehabilitation training, hemiplegia, outcome, stroke

## Abstract

This study explores the clinical effects of Baduanjin rehabilitation training on limb motor function, daily living ability, and quality of life in elderly patients with hemiplegia after stroke. Clinical data of 185 elderly patients with hemiplegia after stroke in our hospital from January 2020 to December 2023 were retrospectively analyzed. According to intervention strategy, patients were divided into observation group (n = 75, Baduanjin rehabilitation training in addition to the conventional training therapy) and control group (n = 110, conventional rehabilitation training therapy). The intervention lasted for 8 weeks. The clinical therapeutic effects, such as National Institutes of Health Stroke Scale scores, modified Rankin Scale score, Barthel Index scores, Fugl–Meyer Assessment scores, 6-minute walking distance, Postural Assessment Scale for Stroke, and Short Form 36 Health Survey scores were compared between the 2 groups. There was no significant difference in baseline data between the 2 groups. The total effective rate of the observation group was significantly higher than that of the control group. After 8 weeks of intervention, the modified Rankin Scale and National Institutes of Health Stroke Scale score of the observation group was lower than that of the control group, while the Barthel Index score was higher, with statistically significant differences. The Fugl–Meyer Assessment scores of the upper and lower limbs of the observation group were significantly higher than those of the control group after 8 weeks of intervention. The 6-minute walking distance and Postural Assessment Scale for Stroke scores of the observation group were significantly higher than those of the control group after 8 weeks of intervention. The mental health, vitality, physical role, and physical function scores of the observation group were significantly higher than those of the control group after 8 weeks of intervention. Baduanjin rehabilitation training has a more favorable clinical therapeutic effective on elderly patients with hemiplegia after stroke, significantly improving their limb motor function, daily living ability, and quality of life.

## 1. Introduction

Acute ischemic stroke (AIS) is one of the leading causes of death and disability worldwide, with significant differences in its incidence and epidemiological characteristics across different regions and populations. According to research, the incidence of AIS has decreased in developed countries, but it is on the rise in developing countries, particularly in Africa.^[1]^ This is closely related to changes in medical conditions, lifestyles, and population structures in these regions.^[2]^ In the United States, stroke is the fourth leading cause of death and the primary cause of disability among adults. In 2009, direct and indirect costs of stroke care in the US were estimated at $68.9 billion, a figure projected to rise with an aging population.^[2]^ In Sweden, from 2010 to 2019, both the incidence of first and recurrent ischemic strokes decreased, with the decline in recurrent strokes nearly double that of first strokes.^[1]^

Management and treatment strategies for AIS and rehabilitation are continuously evolving. Most patients have varying degrees of neurological deficits and residual limb function disorders.^[3]^ Studies show that patients may experience various types of neurological dysfunction after an ischemic stroke, including cognitive impairment, autonomic nervous system dysfunction, and motor dysfunction. These impairments not only affect the patient’s quality of life but can also lead to long-term health issues.^[4]^ While the rehabilitation treatment of neurological dysfunction is very important, rehabilitation plays a crucial role in the treatment of hemiplegia following stroke. Research has found that using force feedback hand rehabilitation robots to assist task-oriented training can further improve the hand function of hemiplegic patients on top of conventional task-oriented training.^[5]^ According to the research on traditional Chinese medicine (TCM), more and more TCM has been proved to have important rehabilitation effects on hemiplegia after stroke.

Ba Duan Jin, as a traditional Chinese qigong therapy, has gradually gained attention in the rehabilitation treatment of patients with hemiplegia after stroke in recent years. Studies show that combining modern rehabilitation techniques with traditional therapies like Ba Duan Jin can promote motor function recovery to some extent and improve the quality of life for hemiplegic patients. Baduanjin can effectively improve patients ‘balance and lower limb function by regulating breathing and body movements. Studies show that balance training has a significant effect on improving motor selection in stroke hemiplegia patients.^[6]^ Through similar mechanisms, Baduanjin may promote patient recovery by enhancing the body’s balance and coordination. A randomized controlled trial examined the impact of Baduanjin on patients with post-stroke cognitive impairment. The results indicated that after 24 weeks of Baduanjin training, patients overall cognitive function, as well as specific cognitive domains such as memory, processing speed, executive ability, attention, and visuospatial skills, all improved.^[7]^ Through systematic evaluation and meta-analysis, studies have found that TCM exercise therapies like Baduanjin can effectively improve cognitive function in the elderly and slow down cognitive decline. In particular, practicing Baduanjin more than 5 times a week for at least 60 minutes each time, over a period of 6 to 9 months, can produce the greatest effect.^[8]^ However, the clinical effective of Baduanjin on hemiplegia after stroke is unknown.

Hence, the present study explores the clinical effective of Baduanjin rehabilitation training on limb motor function, daily life and quality of life in elderly patients with hemiplegia after stroke by a large sample size retrospectively study.

## 2. Methods

### 2.1. Study design and participant selection

We performed a retrospectively study in 247 elderly patients with hemiplegia after stroke in our hospital from January 2020 to December 2023. The study protocol was approved by the 904 Hospital, Joint Logistics Support Force ethics committee (approval number: 2023-109). All study participants provided signed informed consent. Depending on the intervention strategy, patients were divided into an observation group (Baduanjin rehabilitation training in addition to the conventional training therapy, n = 75) and control group (conventional rehabilitation training therapy, n = 110). There were no statistically significant differences in the demographics, such as sex, age, disease history (hypertension, diabetes, hyperlipidemia), smoking, body mass index, admission National Institutes of Health Stroke Scale (NIHSS) scores, and Glasgow Coma Scale scores between the 2 groups (Table 1). Inclusion criteria: confirmed as stroke hemiplegia by cranial computerized tomography or magnetic resonance imaging; all cases were unilateral hemiplegia with standing balance ≥ grade 2; age ranging from 65 to 80 years old; normal communication and interaction; obtained informed consent. Exclusion criteria: patients with malignant tumors; patients with severe abnormalities in vital organs; patients with mental illness or communication and interaction disorders; patients whose condition progressed after 48 hours; previous stroke history.

**Table 1:** Comparison of baseline data.

	Observation group (n = 75)	Control group (n = 110)	*P* value
Age (yr, mean ± SD)	67.10 ± 6.94	68.15 ± 7.32	.329
Gender, no. (%)			.896
Male	43 (57.33%)	62 (56.36%)	
Female	32 (42.67%)	48 (43.64%)	
BMI (kg/cm^2^, mean ± SD)	22.71 ± 1.93	22.37 ± 1.88	.234
NIHSS (before intervention)	11.55 ± 3.15	12.13 ± 2.62	.175
BI (before intervention)	33.75 ± 7.11	34.28 ± 7.63	.634
FMA (before intervention)			
Upper limb	16.11 ± 1.93	16.26 ± 2.05	.618
Lower limb	15.22 ± 2.17	15.39 ± 2.44	.627
6MWD (before intervention)	32.47 ± 5.82	31.97 ± 5.72	.563
PASS (before intervention)	9.91 ± 2.65	10.2 ± 2.74	.475
Risk factors			
Smoking history, no. (%)	19 (25.33%)	24 (21.82%)	.578
Hypertension, no. (%)	48 (64.00%)	72 (65.45%)	.839
Hyperlipidemia, no. (%)	42 (56.00%)	61 (55.45%)	.942
Diabetes, no. (%)	17 (22.67%)	28 (25.45%)	.664
Atrial fibrillation, no. (%)	32 (42.67%)	40 (36.36%)	.388
Living environment, no. (%)			.358
Town	44 (58.67%)	57 (51.82%)	
Countryside	31 (41.33%)	53 (48.18%)	

6MWD = 6-minute walking distance, BI = Barthel Index, BMI = body mass index, FMA = Fugl–Meyer Assessment, mRS = modified Rankin Scale, NIHSS = National Institute of Health stroke scale, PASS = Postural Assessment Scale for Stroke, SF-36 = Short Form 36 Health Survey.

### 2.2. Study protocol

Upon admission, both groups received standard treatment protocols, which included anticoagulation, antiplatelet therapy (aspirin 100 mg/d), and routine neurological care. All patients received 2 weeks butylphthalide treatment, all patients received rehabilitation exercises and meanwhile received hyperbaric oxygen therapy (about 4 wk). The control group participated in conventional rehabilitation exercises, which encompassed scapular girdle movements, continuous turning on both the healthy and affected sides, backward extension stretching exercises for the wrist and ankle joints, alternating between lying and sitting positions, and control training for the hip, knee, ankle joints, and trunk muscles. Furthermore, patients were instructed in sitting and standing balance training as well as walking exercises. In addition to the regimen followed by the control group, the observation group incorporated Baduanjin rehabilitation training, which comprises 8 distinct movements: The exercise regimen consisted of the following movements: “Two Hands Raise to Regulate the Triple Burner,” “Left and Right Open Arrows to Shoot the Eagle,” “Single Raise to Regulate the Spleen and Stomach,” “Look Back to Relieve the Five Labors and Seven Injuries,” “Shake Head and Wag Tail to Remove Heart Fire,” “Two Hands Hold Feet to Strengthen the Kidney and Waist,” “Clench Fists and Glare to Increase Strength,” and “Seven Lifts on the Back to Eliminate All Diseases.” Each movement was executed 3 times, with a 1-minute rest interval between sets. Following the exercise session, participants engaged in a 5-minute rest and relaxation period. The training was conducted 5 days per week. The intensity and speed of the exercises were tailored to the individual conditions of the participants, and the training was continued until each participant reached their tolerance threshold. The intervention period for both groups spanned 8 weeks.

### 2.3. Outcome measurements

#### 2.3.1. Overall clinical efficacy evaluation

Marked improvement: The patient’s neurological deficit score, as measured by the NIHSS, exhibits a reduction of 81% or greater, accompanied by significant enhancements in daily living and motor functions. Effective: The patient’s NIHSS score demonstrates a reduction ranging from 36 to 80%, with corresponding improvements in daily living functions. Futility: The patient’s NIHSS score shows a reduction of <36%, with no observable improvement in daily living and motor functions.

#### 2.3.2. Outcome measurements

After the hospital discharge, patients were followed up by telephone calls once a week. Outcome measurement indicators are as follows:

(1) The NIHSS score was used to evaluate the neurological function of patients before treatment and at discharge. The first endpoint was the modified Rankin scale (mRS), which is used to evaluate patients’ ability to perform activities of daily living 6 months after stroke. The mRS is defined according to patients’ clinical outcomes and has a total score of 5 points. The lower the score is, the better the outcome definition.
(2) Six months Barthel Index (BI) scores were evaluated, Barinette functional deficits correlate with higher BI scores, suggesting an enhanced ability in daily living activities.
(3) Changes in the scores of the simplified Fugl–Meyer Assessment were examined in both groups, with a total possible score of 66 points; higher scores denote superior limb motor function.
(4) Alterations in the 6-minute walking distance and the Posture Assessment Scale for Stroke were also assessed in both groups.
(5) Furthermore, changes in quality of life were evaluated in both groups using the Short Form 36 Health Survey (SF-36), which encompasses 4 scales: mental health, vitality, physical role functioning, and physical functioning, each with a maximum score of 100. Higher scores indicate a better quality of life.

### 2.4. Statistical analysis

Continuous variables were represented as means with standard deviations. Statistical analyses were performed utilizing SPSS software (version 20.0; IBM Corp., Armonk) and GraphPad Prism 6.0 (GraphPad Software, Inc., www.graphpad.com/prism). All data underwent verification by the data committee. Measurements that followed a normal distribution are presented as mean ± SD (x ± s). Quantitative data were evaluated using independent sample *t* tests, while qualitative data were compared using either the chi-square test or Fisher exact test. A *P* value of <0.05 was considered indicative of statistical significance.

## 3. Results

### 3.1. Comparison of overall clinical efficacy

The overall clinical efficacy was evaluated at 6 months between 2 groups, and the total clinical effective rate of the observation group was higher than that of the control group (92% vs 76.37, *P* = .003, Table 2).

**Table 2:** Comparison of the overall clinical efficacy.

	Observation group (n = 75)	Control group (n = 110)	*P* value
Overall clinical efficacy			.003
Marked improvement	39 (52.00%)	38 (34.55%)	
Effective	30 (40.00%)	46 (41.82%)	
Futility	6 (8.00%)	26 (23.64%)	

### 3.2. Comparison of mRS scores

**Figure 1.**
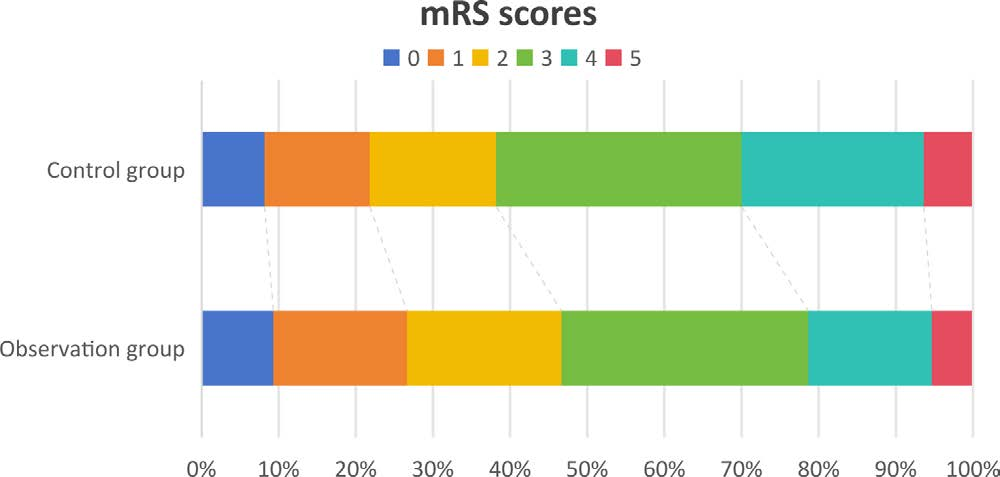
The result of mRS scores between 2 groups.

After 6 months follow-up, we found that there were no significantly difference between 2 groups (*P* > .05, Fig. 1). However, the rate of favorable patients was higher in the observation group than the control group (Fig. 1).

### 3.3. Comparison of NIHSS and BI scores

After 8 weeks rehabilitation, we found that the NIHSS score of the observation group was significantly lower than the control group (*P* < .001), and the BI score was significantly higher than the control group (*P* < .001, Table 3).

**Table 3:** Comparison of the comparison of NIHSS and BI scores.

	Observation group (n = 75)	Control group (n = 110)	*P* value
NIHSS (8 wk)	6.74 ± 1.36	9.27 ± 1.21	<.001
BI (8 wk)	64.28 ± 5.69	48.15 ± 7.48	<.001

BI = Barthel Index, NIHSS = National Institute of Health Stroke Scale.

### 3.4. Comparison of other outcome measurements

After 8 weeks interventions, the Fugl–Meyer Assessment scores of the upper (*P* < .05) and lower limbs (*P* < .05) were significantly higher in the observation group than the control group (Table 4). The 6-minute walking distance (*P* < .05) and Posture Assessment Scale for Stroke scores (*P* < .05) were also significantly higher in the observation group than the control group at 8 weeks evaluation (Table 4).

**Table 4:** Comparison of other outcome measurements.

	Observation group (n = 75)	Control group (n = 110)	*P* value
FMA			
Upper limbs	25.47 ± 2.39	20.46 ± 2.58	<.001
Lower limbs	24.11 ± 2.28	19.72 ± 1.95	<.001
6MWD	107.95 ± 11.27	89.92 ± 9.85	<.001
PASS	26.16 ± 3.27	19.22 ± 3.61	<.001

6MWD = 6-minute walking distance, FMA = Fugl–Meyer Assessment, PASS = Postural Assessment Scale for Stroke.

### 3.5. Comparison of SF-36

In order to comprehensively evaluate the quality of life of the 2 groups of patients, SF-36 scale scores were evaluated. The SF-36 scale scores were significantly higher in the observation group than the control group, including mental health, vitality, role-physical, and physical functioning (*P* < .001, Table 5).

**Table 5:** Comparison of SF-36.

	Observation group (n = 75)	Control group (n = 110)	*P* value
Mental health	67.15 ± 6.82	56.14 ± 5.91	
Vitality	65.19 ± 4.87	54.83 ± 6.12	<.001
Role-physical	73.45 ± 6.71	59.17 ± 7.02	<.001
Physical functioning	70.47 ± 5.39	59.28 ± 6.29	<.001

SF-36 = Short Form 36 Health Survey.

## 4. Discussion

Ischemic stroke is a disease with high incidence, disability rate, and mortality rates worldwide. Studies show that ischemic stroke not only leads to severe physical dysfunction but also significantly increases the risk of death in patients.^[9]^ In high-risk stroke populations in southwestern China, the incidence of ischemic stroke is particularly high, with a total stroke incidence rate of 5.0%, where ischemic stroke accounts for 4.4%.^[10]^ Ischemic stroke not only has a severe impact on patients during the acute phase but can also lead to long-term neurological damage and cognitive impairment. Studies have found that chronic cerebral hypoperfusion can cause cognitive dysfunction.^[11]^ The survey shows that the disability rate of stroke is more than 80%, and about 70% have hemiplegia, which seriously affects the quality of life of patients.^[12,13]^ Post-stroke hemiplegia is one of the common sequelae in stroke patients, severely impacting their daily living abilities and quality of life. Hemiplegia typically manifests as weakness or paralysis on one side of the body, often accompanied by muscle spasms and motor coordination disorders. Studies show that rehabilitation for post-stroke hemiplegia can be carried out through various methods, including physical therapy, medication, and emerging neurorehabilitation techniques.

As a traditional Chinese qigong exercise method, Baduanjin has shown significant effects in the rehabilitation of hemiplegia patients after cerebral infarction in recent years. Studies show that Baduanjin can not only improve the limb function of patients, but also improve the overall physical condition and quality of life.^[7,14]^ A study explored the actual training effects of Baduanjin on patients with hemiplegia after cerebral infarction through semi-structured interviews. The results showed that patients generally believed that Baduanjin could improve limb function and overall physical condition, and most patients were willing to continue practicing Baduanjin. This further validates the positive role of Baduanjin in the rehabilitation of patients with hemiplegia after cerebral infarction.^[15]^ Liu^[16]^ conducted a randomized controlled trial involving 100 patients with post-stroke depression who met the inclusion criteria. These patients were randomly assigned to either the Baduanjin group (n = 50) or the control group (n = 50). The study demonstrated that the combination of Baduanjin exercise and rational emotive behavior therapy significantly improved mood and sleep quality in patients with post-stroke depression. Additionally, it elevated serum levels of 5-HT and BDNF, reduced serum levels of the proinflammatory factor IL-6, alleviated neurological impairment, enhanced daily living abilities, and improved overall quality of life. Furthermore, a systematic review and meta-analysis encompassing 24 studies indicated that Baduanjin could serve as an effective rehabilitation approach for stroke patients, enhancing balance, motor abilities, trunk control, neurological functions, daily living activities, and overall quality of life, while the effectiveness of Baduanjin in enhancing walking ability is inconsistent, necessitating further high-quality randomized controlled trials to validate the findings.^[17]^ In the present study, we also found that rehabilitation training with Baduanjin can improve the efficacy of elderly stroke patients with hemiplegia, reduce neurological deficits and improve daily life, increasing walking distance and improving posture, and improve the quality of life of patients.

In the present study, we found that Baduanjin can indirectly affect motor function by improving balance. Baduanjin has demonstrated considerable advantages in enhancing motor abilities. Previous research on individuals with cognitive impairment following a stroke revealed that Baduanjin exercises greatly enhanced their limb mobility, balance, and walking patterns.^[18]^ This also points to Baduanjin enhancing muscle strength and coordination, along with improving gait parameters like stride length, walking speed, and step frequency. A study revealed that 16 weeks of Baduanjin training led to significant enhancements in patients’ balance, particularly in the Mini-Balance Evaluation Systems Test and the Timed Up and Go assessments.^[14]^ Baduanjin aids in the recovery of post-stroke functional impairments by various means, such as boosting cognitive function, enhancing motor skills, and improving balance. These findings provide a scientific basis for the application of Baduanjin in post-stroke rehabilitation.

The current research had certain limitations. The findings were a single-center retrospective study; it was a retrospective study design, data on certain characteristics could not be found in the files of some patients, and the associated forms of information bias. The single-center approach also warrants consideration as a limiting factor. To validate these observations, it is imperative to conduct large-scale, multi-institutional randomized controlled studies with expanded sample sizes.

## 5. Conclusion

Our research found that Baduanjin rehabilitation training can alleviate limb motor function, daily life and quality of life in elderly patients with hemiplegia after stroke. Nonetheless, our observations lay the groundwork for additional exploration. However, further extensive multicenter randomized controlled studies are necessary to validate these conclusions.

## Author contributions

**Conceptualization:** Hongyan Zhang, Rong Wan, Lihui Zhou.

**Data curation:** Kangping Zhu, Jing Zhang.

**Formal analysis:** Hongyan Zhang, Lihui Zhou.

**Investigation:** Hongyan Zhang, Rong Wan, Lihui Zhou, Kangping Zhu, Jing Zhang.

**Methodology:** Hongyan Zhang, Rong Wan, Lihui Zhou, Kangping Zhu, Jing Zhang.

**Project administration:** Lihui Zhou.

**Software:** Hongyan Zhang, Lihui Zhou.

**Supervision:** Hongyan Zhang, Rong Wan, Lihui Zhou.

**Validation:** Hongyan Zhang, Rong Wan, Lihui Zhou, Kangping Zhu, Jing Zhang.

**Visualization:** Hongyan Zhang, Rong Wan, Lihui Zhou, Kangping Zhu, Jing Zhang.

**Writing – original draft:** Hongyan Zhang, Rong Wan, Lihui Zhou, Kangping Zhu, Jing Zhang.

**Writing – review & editing:** Hongyan Zhang, Rong Wan, Lihui Zhou, Kangping Zhu, Jing Zhang.
